# Supplementary material for: H3K27me3 and EZH Are Involved in the Control of the Heat-Stress-Elicited Morphological Changes in Diatoms
Source: Int J Mol Sci. 2024 Jul 31;25(15):8373. doi: 10.3390/ijms25158373 (PMC11313476; doi:10.3390/ijms25158373)
Supplement: Supplementary file 1 [file ijms-25-08373-s001.zip › ijms-3104800-supplementary.pdf]

Supplementary Material

H3K27me3 and EZH are involved in the control of the heat-stress-elicited morphological changes in diatoms

Mhammad Zarif[1], Ellyn Rousselot[1], Bruno Jesus[2], Leïla Tirichine[1, 3] and Céline Duc[1,*]

[1]Nantes Université, CNRS, US2B, UMR 6286, F-44000 Nantes, France

[2]Institut des Substances et Organismes de la Mer, ISOMer, Nantes Université, UR 2160, Nantes F-44000, France

[3]Institute for Marine and Antarctic Studies (IMAS), Ecology and Biodiversity Centre, University of Tasmania, Hobart, TAS, 7004, Australia

*Correspondence: celine.duc@univ-nantes.fr

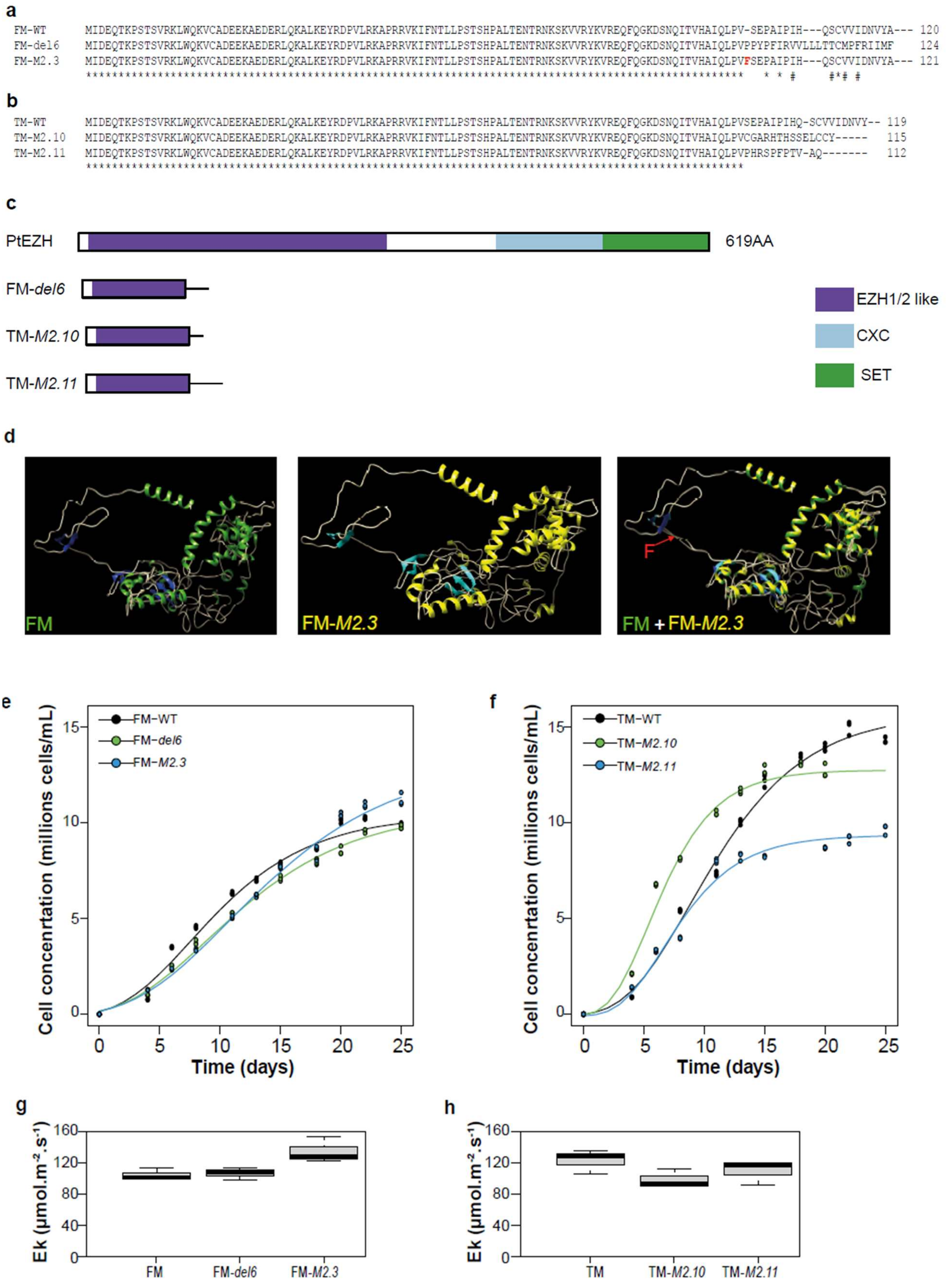

Figure S1. Description of the *Ptezh* mutants in FM and TM lines used in the study. (**a-b**) Protein sequence alignment of putatively produced proteins from the *PtEZH* gene in FM (a) and TM (b) lines. The complete protein produced by the

FM-*del6* mutant is presented. The insertion of the F residue in FM-*M2.3* is indicated in red. Asterisks indicate a fully conserved residue and the hash conservation between residues with either strong or weak similar properties. (**c**) Functional domains of the PtEZH proteins putatively produced in *Ptezh* mutants. Each domain is depicted at its position by a different color and the code is indicated at the right. CXC, C-X(6)-C-X(3)-C-X-C motif. (**d**) Ribbon representation of PtEZH proteins produced in FM and putatively in FM-*M2.3*. The insertion of the F residue is indicated by a red arrow. Models were generated with Phyre2 (Kelley et al., 2015). Superimpositions were done with the Chimera software. (**e-f**) Growth curves of wild type and *Ptezh* mutants in FM (e) and TM (f) lines. (**g-h**) Photosynthesis analysis presenting the Ek parameter in FM (g) and TM (h) lines. Ek is the photon irradiance at onset of light saturation coefficient. Student's t test; *P < 0.05.

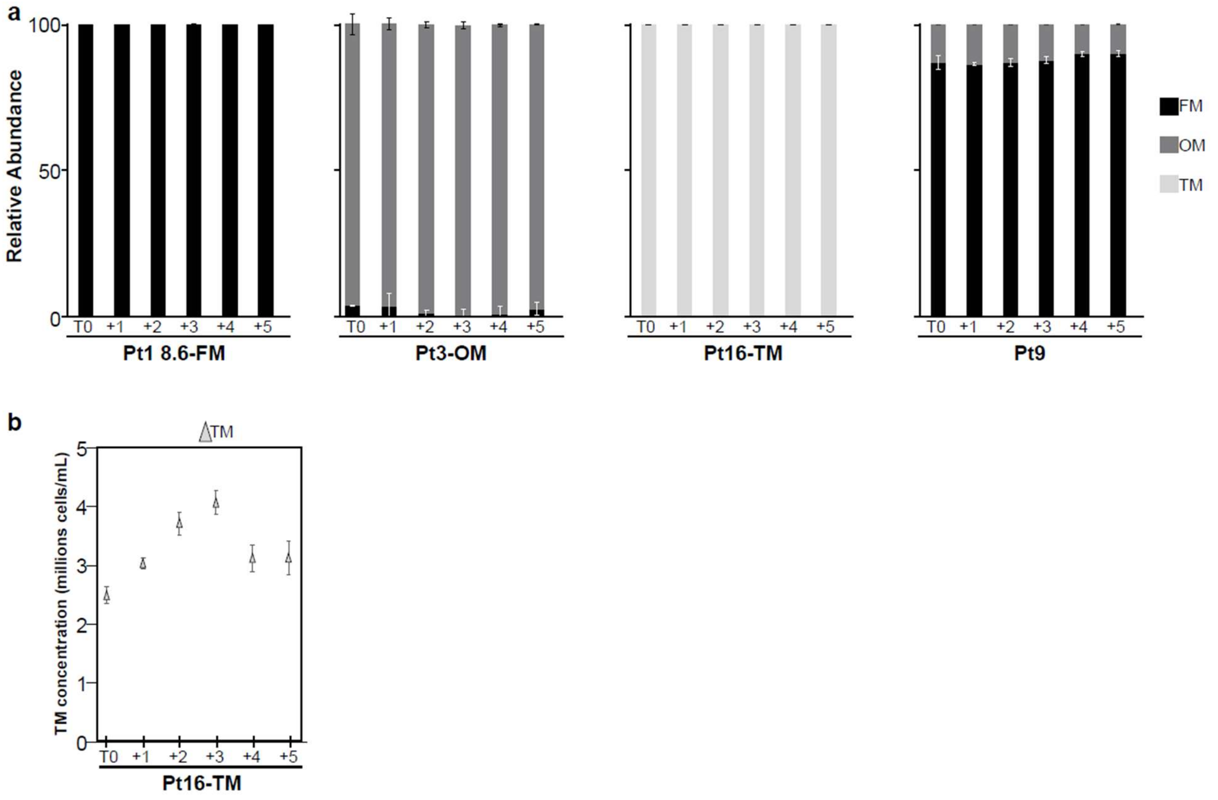

Figure S2. Control for the prolonged-heat stress experiments carried out on representative ecotypes of *P. tricornutum*. (**a & c**) Relative abundance of each morphotype in Pt1 8.6-FM, Pt3-OM and Pt16-FM representing FM, OM and TM morphotypes, respectively. The Pt9 tropical strain was used as a control. For MHS, the 7-day-old cultures pre-acclimated at 19°C were maintained at 19°C for an additional 5 days as a control (a). For EHS, the 5-day-old cultures pre-acclimated at 19°C were maintained at 19°C for an additional 2 days as a control (c). (**b**) Cell concentration measured for Pt16 subjected to MHS in which no morphotype change was observed.

a

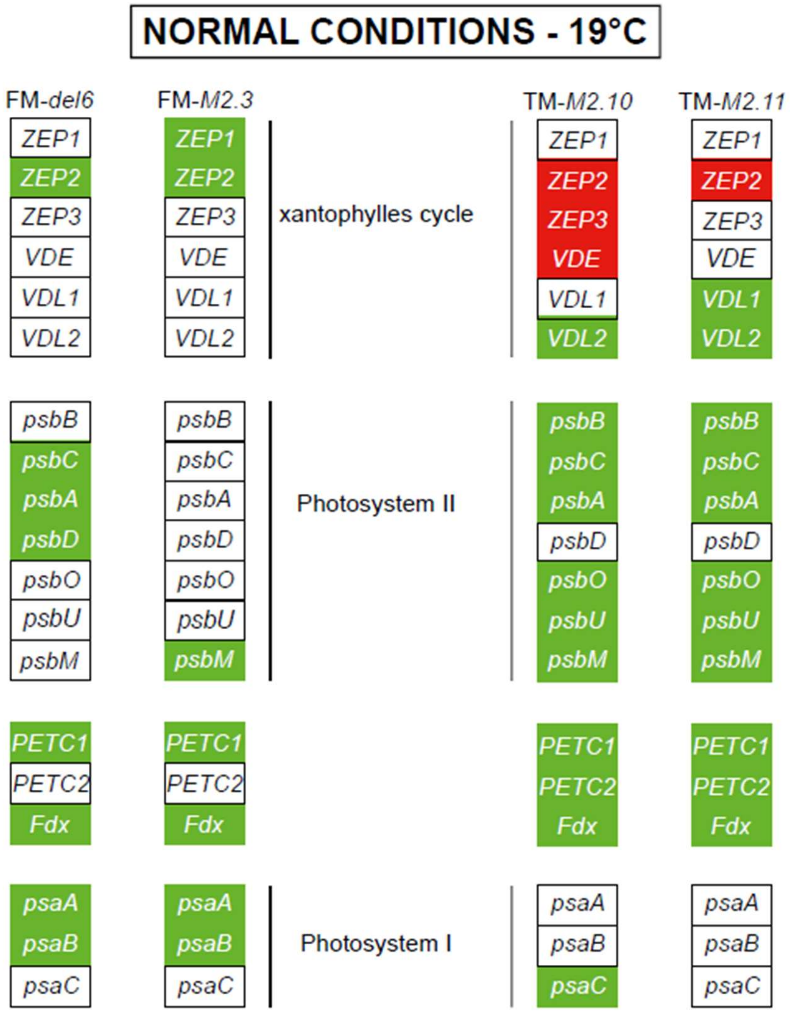
NORMAL CONDITIONS - 19°C
FM-del6
FM-M2.3
TM-M2.10
TM-M2.11
ZEP1
ZEP2
ZEP3
VDE
VDL1
VDL2
xantophylles cycle
psbB
psbC
psbA
psbD
psbO
psbU
psbM
Photosystem II
PETC1
PETC2
Fdx
psaA
psaB
psaC
Photosystem I

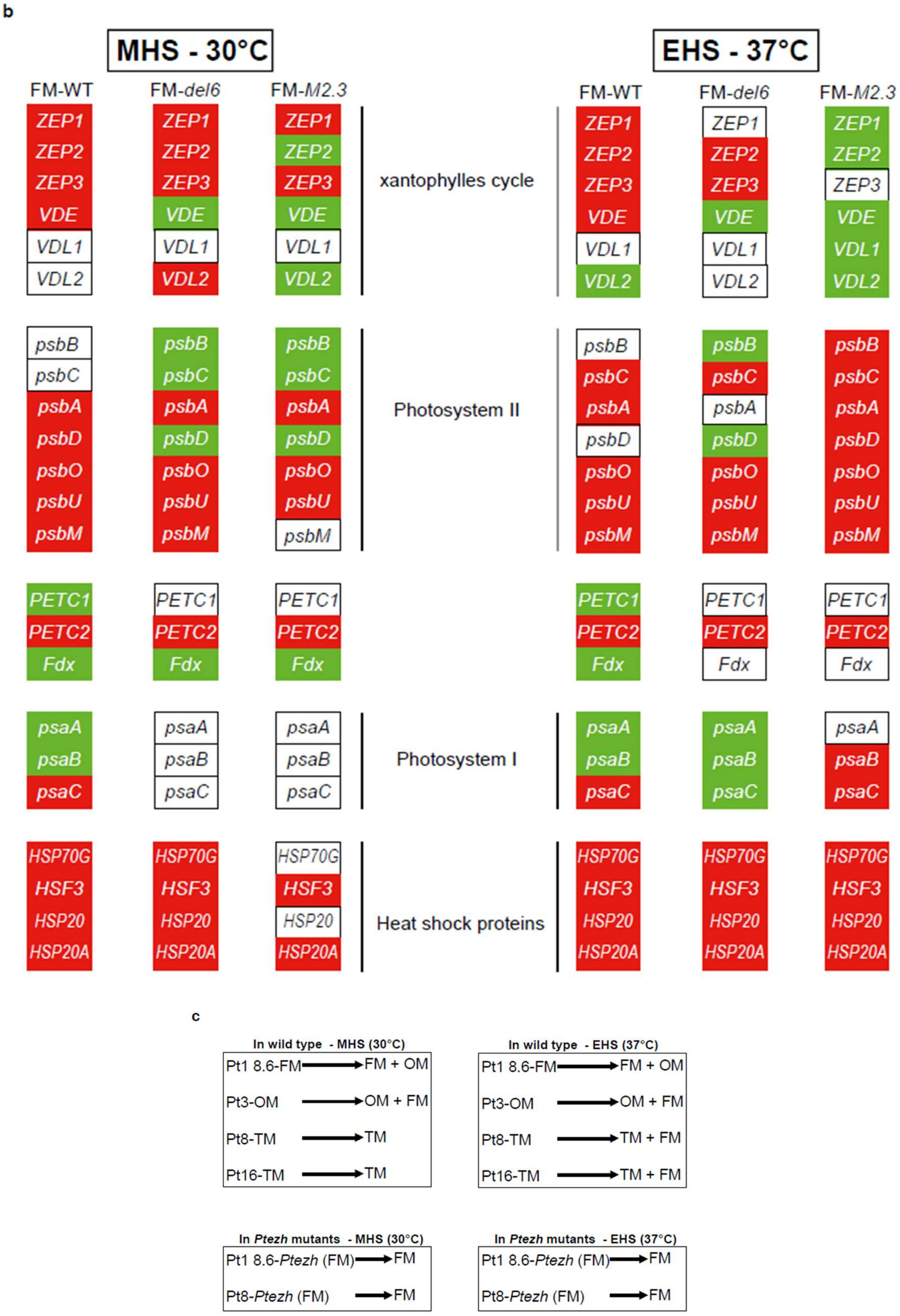

Figure S3. Schema representing changes in transcript abundances monitored by qRT-PCR in standard growth conditions (19°C) and upon heat stress (MHS and EHS) as well as heat-elicited morphotype changes. (**a**) Transcript

abundance changes for genes encoding enzymes involved in the xanthophyll cycles and photosynthesis in *Ptezh* mutants in FM (FM-*del6* and FM-*M2.3*) and TM (TM-*M2.10* and TM-*M2.11*) lines. Changes corresponded to levels measured by qRT-PCR on three biological replicates consisting of 5-day-old cultures at 19°C presented in Fig. 2 (xanthophyll cycles) and Fig. 3 (photosynthesis). (**b**) Transcript abundance changes for genes encoding enzymes involved in the xanthophyll cycles, photosynthesis and heat shock proteins in wild type (FM) and *Ptezh* mutants (FM-*del6* and FM-*M2.3*) upon heat stress. Changes corresponded to levels measured by qRT-PCR on three biological replicates consisting of 7-day-old cultures pre-acclimated at 19°C transferred to 30°C for 4 days (MHS) and 5-day-old cultures pre-acclimated at 19°C transferred to 37°C for 2 days (EHS) presented in Fig. 8 (MHS) and Fig. 9 (EHS). The Zeaxanthin Epoxidase ZEP1, ZEP2 and ZEP3 enzymes putatively convert (i) zeaxanthin *via* the antheraxanthin intermediate to violaxanthin and (ii) diadinoxanthin in diatoxanthin. The Violaxanthin De-Epoxidase VDE, Violaxanthin De-epoxidase-Like VDL1 and VDL2 enzymes putatively catalyze the reverse reactions [10]. For photosynthesis, analyzed genes encode proteins from PSII (antenna: PsbB and PsbC; reaction center: PsbA and PsbD; OEC: PsbO and PsbU; small transmembrane protein: PsbM), the cytochrome b6-f complex (Rieske proteins: petC1 and petC2), ferredoxin (Fdx) and PSI (trans-membrane subunits: PsaA, PsaB; stromal subunit: PsaC). For heat shock proteins, analyzed genes encode heat shock proteins (HSP, Heat Shock Protein; HSF3, Heat Shock Factor protein 3). Green and red squares correspond to genes with increased and decreased transcript levels, respectively. Black squares correspond to genes with no changes monitored in transcript levels. (**c**) Heat-elicited morphotype changes monitored in the various morphotypes. In Pt1 8.6-FM, OM cells appear in response to MHS (Fig. 4a) and EHS (Fig. 4c). In Pt3-OM, FM cells appear in response to MHS (Fig. 4a) and EHS (Fig. 4c). However, FM cells appear in Pt16-TM in response to EHS (Fig. 4c) but not in MHS (Fig. 4a). The Pt1 8.6-*Ptezh* mutants (FM-*del6* and FM-*M2.3*) display FM cells and no morphotype changes were observed in response to MHS (Fig. 6a) and EHS (Fig. 6c). The Pt8-*Ptezh* mutants (TM-*M2.10* and TM-*M2.11*) display FM cells and no morphotype changes were observed in response to MHS (Fig. 6a) and EHS (Fig. 6c). MHS corresponds to 7-day-old cultures pre-acclimated at 19°C transferred to 30°C for 4 days. EHS correspond to 5-day-old cultures pre-acclimated at 19°C transferred to 37°C for 2 days (EHS)

Table S1: List of primers.

Primer Name	Primer sequence (5'-3')	Gene Name	Gene ID	qPCR Efficiency
RPS_qpcr_For	CGAAGTCAACCAGGAAACCAA	*RPS*	Phatr3_J10847	1.73
RPS_qpcr_Rev	GTGCAAGAGACCGGACATACC			
TBP_qpcr_For	ACCGGAGTCAAGAGCACACAC	*TBP*	Phatr3_J10199	1.88
TBP_qpcr_Rev	CGGAATGCGCGTATACCAGT			
HSP20_qPCR_F	GGTAGAGTCGAAGGCGGATG	*HSP20*	Phatr3_J54656	1.93
HSP20_qPCR_R	GTCGAGATCTCAAGACCGTCC			
HSP20A_qPCR_F	CGCTTTACGATCGGTGACAAC	*HSP20A*	Phatr3_J35158	1.95
HSP20A_qPCR_R	TCTTTCGCCTTTTTGGGTGC			
HSP70G_qPCR_F	CAAGAAACCCAAGCCCAAGC	*HSP70G*	Phatr3_J55122	1.84
HSP70G_qPCR_R	GTCCTCCATAGCGTCTGTCG			
HSFB3_qPCR_F	GCCACCAACACCCTTTTTCC	*HSF3*	Phatr3_J48361	2.06
HSFB3_qPCR_R	GTTACTGGCTGTCCCCCTTG			
psbB_qPCR_F	ACTGTACGTCGTATGCCAGC	*PsbB*	YP_874387.1	1.92
psbB_qPCR_R	GATTCTGCACGACGGAATGG			
psbC_qPCR_F	TCACTGGTGGCATTCAGGTC	*PsbC*	YP_874376.2	1.86
psbC_qPCR_R	AGGACGCATCGATAACACAGG			
psbA_qPCR_F	GCTGACATCATCAACCGTGC	*PsbA*	YP_874444.1	1.95
psbA_qPCR_R	TGGAGCTGTTAAAGCAACTGG			
psbD_qPCR_F	GGTATTCGTGCTTGGATGGC	*PsbD*	YP_874377.1	1.89
psbD_qPCR_R	TTCCACGTGGCAATACCTCC			
PsbO_qPCR_F	AGCTGTCCGACACCGATATG	*PsbO*	Phatr3_J20331	1.91
PsbO_qPCR_R	CACGGGCGTAGAAGATACCC			
PsbM_qPCR_F	TAGGAACCCTCCTTCCCTGC	*PsbM*	Phatr3_J55057	1.96
PsbM_qPCR_R	CTCGGCATCCTGTCCACC			
PsbU_qPCR_F	TCCCGGGATTGTCTGGAAAG	*PsbU*	Phatr3_J26293	1.96
PsbU_qPCR_R	TAGAGGCCGTTGTTGAAGCG			
PETC-1_qPCR_F	TTGTCACGGGTCCCAATACG	*petC1*	Phatr3_J46657	2.07
PETC-1_qPCR_R	TCCGACCAAAGACCTAAGCG			
PETC-2_qPCR_F	TCCTCTTCCTCTCGCCCTC	*petC2*	Phatr3_J13358	2.08
PETC-2_qPCR_R	TTCTCCGGTACGGAAATCGC			
psaA_qPCR_F	TGCTCCTGCAATCCAACCTC	*psaA*	YP_874359.1	1.95
psaA_qPCR_R	CACGAGCTAAGAAGAACGCC			
psaB_qPCR_F	ATTTGGGGTGGTAACCCAGG	*psaB*	YP_874358.1	2.07
psaB_qPCR_R	AATAGTCACGTAGCCAGCCC			
psaC_qPCR_F	GCGTGTCGAAGATTGTGTAGG	*psaC*	YP_874487.1	2.12
psaC_qPCR_R	TTCTAGTCGTTTCTGCACCC			
Fx_qPCR_F	TTAGATCTCGCGTTCGAGCC	*Ferredoxin*	Phatr3_Jdraft1610	2.02
Fx_qPCR_R	TGATCCGTTTCCAGAAGCCC			
AtpB_qPCR_F	ATTCAAGCTGTATACGTACCAG	*AtpB*	YP_874407	2.05
AtpB_qPCR_R	TCTAAATGTGCGAATGTTGTCG			
ZEP1_qPCR_F	CGCAAACGTATTCCCCGAAC	*ZEP1*	Phatr3_J45845	2.00
ZEP1_qPCR_R	AAAAATGGCCCAACCAACGG			
ZEP2_qPCR_F	ATGCCGAAGCTGTGTTTGAG	*ZEP2*	Phatr3_J5928	1.89
ZEP2_qPCR_R	TGGGGACAATTCTACTTCCGC			
ZEP3_qPCR_F	TGTCACCAAAATACTCCAGC	*ZEP3*	Phatr3_J10970	1.80
ZEP3_qPCR_R	AACGCCTTGAAGTCGATCTG			
VDE_qPCR_F	TTCCATCAAGGCGCAAAAGC	*VDE*	Phatr3_J51703	2.15
VDE_qPCR_R	TGCTGGGAGGTTTCTCGTTC			
VDL1_qPCR_F3	AAGGCGGGCTTGGATTTTG	*VDL1*	Phatr3_J36048	1.96
VDL1_qPCR_R3	GCTTGTGCATCGTTCAAAGAG			
VDL2_qPCR_F2	ATACTACAACGGCAAAACCC	*VDL2*	Phatr3_J45846	1.81
VDL2_qPCR_R2	CTTCCTTGGCAATGGAGTAG			
